# The Third Session of the Research Society of the American Red Cross in France

**Published:** 1918-03

**Authors:** 


### RESEARCH SOCIETY REPORTS

### THE THIRD SESSION OF THE RESEARCH SOCIETY OF THE AMERICAN RED CROSS IN FRANCE

February 15 and 16, 1918, at 6, rue Piccini, Paris.

The meeting was called to order by Major Lambert, Chief Surgeon of the American Red Cross in France, who introduced Captain Walter B. Cannon, Chairman of the Research Committee of the American Red Cross in France, as the presiding officer for the Session. The subject of the first meeting was Cerebro-Spinal Meningitis.

A paper by PROFESSOR DOPTER of Val-de-Grâce was read in translation by Major Lambert. It was entitled " Observations on Sero-Therapy for Cerebro-Spinal Meningitis. "

Professor Dopter stated that he had demonstrated as early a. 1909 that antimeningococcic serum was powerless against one of the organisms similar to the meningococcus, which he isolated and termed the parameningococcus. In 1914 together with M. Pauron, he differentiated three other varieties. All of these resisted the curative action of the serum.

Since, however, the cases due to the meningococcus proper were far more numerous than the others, and since the serum against this organism had more than established its curative value, Professor Dopter saw no reason for attempting to prepare a polyvalent serum against the original meningococcus and the various types of parameningococcus as well, thereby running the risk of weakening its effect against the more dangerous meningococcus. He did, however, produce a polyvalent serum against the A, B, and C types of the parameningococci, which he administered in cases in which the presence of the parameningococci could be proved. Up to the outbreak of the war 1073 patients were treated along these lines at the Institut Pasteur, with a mortality of 255 or 23.76 o/o.

In 1916 the parameningococcic varieties began to appear more frequently. A polyvalent serum[1] was therefore prepared which would be effective against the parameningococcic types and the meningococcus as well. This was employed in 451 cases with a mortality of 125 (27.7 o/o).

In 1917 the parameningococcic types were still frequent, and the use of the polyvalent serum was continued in 409 cases with a mortality of 130 (31.78 o/o).

It would therefore appear that the polyvalent serum has, if anything, increased the death-rate. This result also confirms the conclusions reached by M. Louste in the region of the interior.

Professor Dopter does not believe, however, that the use of the monovalent serum should be resumed, because the varieties of the parameningococci are now found in about 45 o/o of the cases as against 3 o/o or 4 o/o observed before the war. If the use of a parameningococcic serum is delayed until the laboratory can demonstrate the presence of the parameningococci, a high mortality is sure to result.

There is now general agreement that two types of organism causing the disease are far more common than the rest : these are meningococcus A (the typical meningococcus) and meningococcus B (Dopter's parameningococcus A). Meningococcus C (Dop-

1. This serum was composed of a mixture of 2 parts of anti-meningococcus serum, 1 part of anti-parameningococcus serum, and 2 parts of serum from animals immunized simultaneously against both organisms.

ter's parameningococcus B) is relatively rare. For the present, therefore, it has seemed best to employ in all cases a mixed bivalent serum against these two organisms from the start. Monovalent sera have been prepared, however, against the A, B, and C types, for use in case the laboratory demonstrates any one of these types as the causal agent. In this respect Professor Dopter states that he has come to the same conclusion as that first reached by Professor Netter and later by MM. Nicolle, Debains, and Jouan. The Institut Pasteur furnishes the bivalent serum under the name of polyvalent serum, and furnishes the monosera as well. So far the A + B serum has given promising results, and in the cases where it has been used there has been no need of resorting to the monovalent sera. Professor Dopter, however, calls attention to the following points which seem to him highly important :

1. Probably the activity of the bivalent serum is not so great against any organism as a specific monovalent serum would be. The death-rate, therefore, may be slightly raised above the normal by its use in place of a monovalent serum. This increase, however, is probably less than would result from the delay in ascertaining the type of the organism before administering the serum.

2. The closest collaboration between the clinician and laboratory worker should be maintained in spite of difficulties. It is often true that laboratory tests fail to give desired results, and in these cases the clinician must be guided by the results obtained by his treatment in deciding upon the serum to be used.

3. The present method of treatment may not survive the war, for the parameningococcic group of organisms seems to have come into importance with the war, and it may die out with it.

4. In cases of epidemic, for which one of the types has been shown to be responsible, treatment should begin with the specific monovalent serum for the organism in question, but laboratory tests should be made to guard against any aberrant cases.

Professor Arnold Netter read a report on 52 cases of cerebrospinal meningitis, setting forth the results of his own study and that of Professor Nicolle. It was Professor Nicolle's belief that an efficient serum for the cure of this disease must be bivalent against types A and B of the meningococci, which are the types responsible for the disease in the majority of cases. It was possible to obtain such a serum.

Meningococci were identified by cultures in 33 out of the 52 cases. In 22 of these they were of the type B and in 11, of the type A. Types C and D were not found at all. It appears that type B is related in some special manner to septic or generalized forms of the disease.

Out of the 22 cases of type B, 8 were accompanied with purpura and 3 with rashes. Before 1915 out of 181 cases purpura was observed in only 5 cases. At that time meningococci were demonstrated in the purpuric lesions[1]. Purpura was much less common in cases of type A.

The following table showed the increased tendency during the last few years for meningitis to be accompanied with extra meningeal determinations:

| | Number | Purpura | Arthritis | Iridocyclitis | Endocarditis |
|---|---|---|---|---|---|
| 1908. . . . . . . . | 3 | — | 1 | — | — |
| 1909. . . . . . . . | 81 | 2 | 3 | 1 | — |
| 1910. . . . . . . . | 29 | — | — | — | — |
| 1911. . . . . . . . | 16 | 1 | 1 | — | — |
| 1912. . . . . . . . | 10 | — | — | — | — |
| 1913. . . . . . . . | 21 | 1 | 1 | — | — |
| 1914. . . . . . . . | 21 | 1 | 3 | 1 | — |
| 1915. . . . . . . . | 67 | 9 | 7 | 2 | 2 |
| 1916. . . . . . . . | 42 | 6 | 6 | 1 | 2 |
| 1917. . . . . . . . | 52 | 14 | 6 | 3 | — |

This same tendency to extra meningeal determinations has also been observed in Great Britain and Germany.

Since type B is more common than type A, a type of serum should be used from the first day of the treatment which is as efficient against type B as against type A. This object may be attained either by mixing monovalent sera against A and B or by the use of a bivalent serum prepared from horses immunized against types A and B. The quantity of the efficient dose of bivalent serum is only half that of the mixed sera. Such a bivalent serum was prepared by Professor Nicolle and his colleagues in September, 1917. 11 cases have since been treated, 3 of type A, 6 of type B, and 3 not identified. There were 3 deaths, 2 on the day of the first injection and 1 due to secondary infection. In all the cases in which the serum might be expected to succeed, recovery was complete. In only 2 of these cases was it necessary to make more than 3 injections.

In 5 cases Flexner's serum was used. Of these 2 were of type A, 2 unidentified, and 1 of type B. There was 1 death from type A and 1 from the unidentified cases.

1. Netter. Les formes purpuriques de la méningite cérébrospinale, *Revue de médecine*, Jan., 1917; *Soc. de biol.*, July 22 and Nov. 18, 1916, and June 30, 1917, and *Soc. des hôp. de Paris*, July 28, 1916.

In 4 cases a monovalent serum against type A was used. Of the 3 cases cured, 2 were identified as type A and 1 was unidentified. The only death was in the case identified as type B.

The impressions gained from the study of the 52 cases here presented confirms the belief entertained by Professor Netter for nine years that it is necessary to discriminate between types of meningococci.

LIEUTENANT COLONEL GORDON, R. A. M. C., spoke on cerebro-spinal meningitis from the viewpoint of prevention, describing bacteriological investigations made during outbreaks in the last three years among the British troops in England. Only last year was it possible to devote much time to the attempt to secure increased potency of therapeutic serum.

In 1915, cerebro-spinal fever broke out to an alarming extent among the recruits in training in England. In order to check the spread the War Office had the cases and their contacts isolated. A person was considered a contact if he had been within three yards of the case under conditions favorable to infection. The naso-pharynx of each contact was swabbed. Those contacts in whom no suspicious organisms were found were dismissed. The rest were tested repeatedly until it was certain that the meningococcus was absent.

For the purpose of making these observations, a central laboratory was established in London and district laboratories throughout the country. It was found that 70 o/o of the contacts could be returned to duty within 48 hours. The meningococcus was identified by subculturing suspicious colonies on to two slopes of solid medium free of blood or hematin. One was placed at 37° C. and the other at 23° C. If within 48 hours the suspected organism had grown at 37° C. but not at 23° C., it was regarded as the meningococcus. Sugar reactions were employed for confirmation.

*Differentiation of the Meningococcus by the Agglutination Test.* — At first the antimeningitis serum failed to agglutinate. It was therefore necessary to begin *ab initio* and to define the characters of the coccus of the outbreak. The speaker, working with Captain Murray, submitted 32 meningococci isolated from the spinal fluid of cases to the agglutination test checked by the absorption test. They prepared within 10 days by an intensive method and a process of saturation, an agglutinating serum with a sufficient titer for the purpose. A rabbit was immunized against the first of the meningococci, and then all of the 32 were tested by his serum. Of them, 19 proved identical to the meningococcus in question. In the same manner three other types were differentiated with the following distribution :

| | | | | |
|---|---|---|---|---|
| Type . . . . . . . . . . . | 1 | 2 | 3 | 4 |
| Specimens . . . . . . . . . | 19 | 8 | 4 | 1 |

Since the original tests 350 meningococci from cerebral fluid have been examined at the laboratory, with the result that 98 o/o of them have been found identical with one or another of the four groups. Those in the third and fourth groups were found in some instances to show affinities with the larger and more important first and second groups, but they were clearly distinguished by the absorption test. In the case of type 2, three sub-groups have been distinguished, but all of the three are included by an agglutinating serum prepared against a representative specimen of the group. Type 1 is most frequently observed at the beginning of an epidemic, type 2 is frequently observed later, and types 3 and 4 when the epidemic is on the wane. Cases of type 1 are usually the most severe, although fulminating cases with any of the types may occur. Type 1 apparently represents the normal meningococcus, and type 2 the parameningococcus.

*Inter-relation of the Types.* — Agglutination tests, controlled by the absorption test, point clearly to the fact that these various types are not merely temporary variants of one and the same microorganism, but that they are specifically distinct, though closely allied, in a manner similar to the typhoid bacillus and paratyphoid A and B. Superimposition tests were carried out upon rabbits to confirm these results. It was argued that if a rabbit already in course of elaborating agglutinin to a given type of meningococcus, received a superimposition of another type, the animal would merely elaborate a larger amount of the same agglutinin, provided the type superimposed were merely a variant. If, however, the type superimposed were specifically distinct from the first, a new agglutinin specific to the new coccus, and previously absent, would be elaborated. A new agglutinin specific to each of the 4 types actually appeared in the rabbit's blood in these superimposition tests. Thus the conclusions of the absorption test were confirmed, indicating that the types are specifically distinct.

Immunity tests were carried out. Four groups of rabbits were immunized, each against a separate type. Different rabbits from each group and controls were then saturated with living cultures of the various types. It was found that in the cases of types 2, 3, and 4, only those rabbits survived which were prepared with the specific coccus with which the test was made. In the case of type 1, however, the homologous rabbit appeared, if anything, to be less resistant than a normal animal. It would then appear that immunity afforded against types 2, 3, and 4, is monotypical in character, and that immunization against type 1 is far more difficult, perhaps because its endotoxin is more powerful than that of the other types.

Major Hine has produced a univalent agglutinating serum for the identification of each of the types. It has been used for two and a half years with the following results :

It has been found that a case of cerebro-spinal meningitis carries only a single type of the meningococcus, and, at the onset, the same type is present in the nasopharynx and in the cerebro-spinal fluid when these are positive. This fact has proved especially helpful in doubtful cases when the fluid has failed to show the meningococcus. Carriers also have been found to be univalent as to type throughout the carrying period.

Furthermore it has been observed that the carriers found in camps and barracks show the types in the same proportion as the cases. If the cases are due mainly to type 2, the majority of the carriers found in the vicinity have also been found to have the same type. A similar relationship has been observed with regard to the carrier rate and the case rate. Observations made by Captain Flack and Captain Glover[1] showed that a sharp recrudescence of the disease was preceded by a rise in the carrier rate. At such times the number of carriers is greatly increased. In one instance there was a carrier rate of 70 o/o in a part of the camp where no cases had occurred. Non-contacts, indeed, may sometimes show a higher carrier rate than those usually considered to be contacts.

Captain Glover found also that high carrier rates in his district were associated with over-crowding in the sleeping quarters. When the beds were distanced the carrier rate fell in the course of a few weeks from over 20 o/o to the normal rate of 5 o/o or less.

*Treatment of Carriers.* Although the chronic carrier may be responsible for perpetuating the disease, the temporary carrier is apparently as important in creating and maintaining an epidemic. It was therefore believed that any form of disinfecting which would reduce the abundance of the meningococcus in the nasopharyngeal secretion ought to be of value. Experiments were carried out at the Central Laboratory to ascertain the disinfecting power of air densely charged by a steam atomizer with such sub-

1. *R. A. M. C. Journal*, Jan. 1918.

stances as zinc sulphate and chloramine. It was found that air charged with droplets of 1 o/o zinc sulphate, or 2 o/o chloramine could be easily tolerated, and that it temporarily destroyed or reduced the meningococci in the nasopharynx of carriers, provided that the men inhaled the sprayed air vigorously for a period of from 5 to 10 minutes. Chloramine has been dropped because it has been found too trying. The portable Levick sprayer has been used with good results upon carriers of meningitis, and is now being tried against influenza, measles, and other diseases transmitted by the upper respiratory passages. An improved form of sprayer has been elaborated by Major Hine, which eliminates the heat and fumes given off by the portable sprayer, and makes it possible to handle a much larger number of men together.

The problem of chronic carriers is more difficult. Acriflavine (1 : 1000) applied several times daily appears to be the best treatment. Such carriers should be specially examined by a rhinologist for possible abnormalities.

*Measures desirable for the prevention of Cerebro-Spinal Fever.* — As is not the case in civil practice, the character of the disease as a mass infection is the point of prime importance in war. The coincidence of the catarrhal and cerebro-spinal seasons is another important consideration. The meningococcus is air-borne and spreads chiefly at night. The striking distance is short.

In the light of these observations, Captain Glover's discovery with regard to over-crowding and the carrier rate, is chiefly to be considered in any attempt to prevent the spread of the disease. Men should be given as much floor space as possible; according to Captain Glover, at least 2 1/2 feet between beds. At least, free ventilation should be provided. If over-crowding is unavoidable, it is advisable to have a steam spray available for nasopharyngeal disinfection.

At the time of the outbreak preventive measures should be most active. Not only contacts, but, if possible, the whole camp or unit should be swabbed, and the carriers isolated. Whenever these measures have been carried out, the disease has invariably come to an end in the particular unit affected. When the meningococcus is widely diffused, this method is naturally impracticable. The carrier-rate, however, may be used as a guide. Representative non-contacts as well as contacts should be swabbed. If the general carrier-rate is thus found to be above 20 o/o, spacing should be insisted upon, and the ideal procedure would be to spray all the men in the camp or unit for 5 or 10 minutes daily until the carrier rate falls to normal.

Any attempt to deal with chronic carriers is best carried on in the autumn when the general carrier rate is at its lowest figure.

*Therapeutic Serum.* — At the end of the first year of the war, a serum of very high potency was available. Out of 33 cases treated by this serum in the London District within three days of the onset, no less than 30 recovered. When the supply of this serum was exhausted a second supply procured from the same source proved much less effective clinically.

An attempt has therefore been made to standardize the serums. By applying a modification of Besredka's method of extracting endotoxin to virulent specimens of the various types of meningococcus, which had first been dried, it was found possible to grade the serum according to its anti-endotoxic value. The serum that had given the best results clinically was found to contain the greatest amount of antiendotoxin to each of the two commonest types of the meningococcus.

At the present time, therefore, it is possible to have serum of assured potency, in regard not only to opsonin, but also to antiendotoxin. It contains enough of the latter in 0.5 cc. to neutralize 1 M.L.D. of endotoxin of each of the two commonest types of the meningococcus. Individual horses also are prepared against individual types of the organism, and their sera pooled, so that a multivalent serum for all of the types is obtained. The proportions are : 40 o/o type 1, 40 o/o type 2, 10 o/o type 3, and 10 o/o type 4. This mixture is employed at the start. Monovalent sera are also available for cases that fail to respond to the pooled serum.

The clinical results have been most satisfactory. All of the 17 cases infected by type 1 meningococcus, treated by the pooled serum, during the present year, have recovered. In most of them 2 doses were sufficient. Although the disease has not been very severe this year, the speaker considered these results very encouraging.

Major Michael Foster, R.A.M.C., gave an account of his experience in dealing with the clinical aspect of the disease. He said that during the first three winters of the war he had charge of cases of cerebro-spinal meningitis, occurring in a large military area, treating about 150 authentic cases, besides about 200 suspected cases.

The first lesson from the experience was the confirmation of the point on which all now agree, that clinical symptoms cannot be relied upon for an accurate diagnosis. Bacteriological tests alone are trustworthy. Although American physicians are more inclined to perform lumbar punctures than the British, it is at least necessary to know the symptoms which indicate lumbar puncture.

One must dismiss from one's mind anything like a type case presenting all the classical symptoms. When they all appear diagnosis has failed to be a helpful forecast since the disease has become firmly established. Even symptoms which might be expected early often appear late. For this reason the absence of any one symptom should not lead one to defer puncture. Rigidity is one of the earliest symptoms. Head retraction and stiffness of the neck are late symptoms, although the stiffness of the neck may precede head retraction by a considerable interval of time. A virulent spinal fluid, however, may be obtained before there is any stiffness at all. Hence the absence of these symptoms does not contra-indicate lumbar puncture.

There are about four forms of rash typical to the disease. The true purpuric rash in conjunction with virulent cases is unmistakable. The second form is a reddish, small, papular rash that comes out on points of pressure at a very early stage, perhaps the first or second day, or, at the latest, on the third day. This may be taken as a reliable sign indicating lumbar puncture. An erythema may also appear on the first or second day. On the third or fourth day a rash, which the speaker considered very typical, may appear with a definite distribution. It occurs on the dorsum of the hands, the surface of the arms, on the abdomen, on posterior and anterior parts of the shoulders, and on the dorsum of the foot. It varies in size from that of a No. 1 shot to that of a split pea and varies in colour from a delicate geranium shade to that of a ripe grape. It may form larger maculae. Although it usually appears on the third or fourth day, it may appear earlier and cause confusion.

Two points are of special importance in early diagnosis : Kernig's sign and retention of urine, or difficulty in micturition. Kernig's sign usually comes out within from 15 to 24 hours after the onset. It is one of the earliest symptoms of all, and has come to be regarded as a critical diagnostic point. Although it occurs in a great number of cases it may be absent entirely, especially in fulminating cases in which death may ensue within 24 hours. In some cases Kernig's sign does not develop until the third or fourth day.

To illustrate the danger of waiting for diagnostic indications, the speaker referred to an epidemic of the disease in a badly infected camp. There were many suspected cases with nothing the matter, but many others with anomalous developments. One man was sent in with no temperature, no headache, no vomiting. The only trouble seemed to be a considerable difficulty in passing water. He had no stiff neck and no Kernig's sign. He seemed a little strange, however, and very nervous. A puncture revealed a virulent fluid swarming with meningococci. The man subsequently developed Kernig's sign and died. It is therefore clear that Kernig's sign cannot be relied upon to give an early indication for puncture.

The macular rash may sometimes appear so early as to be confusing. Another case from the same camp came in with an almost universal macular rash, but no headache or vomiting, and no Kernig's sign. He was punctured, but a perfectly clear fluid was obtained with an absolutely normal cell count. During the night he collapsed and became unconscious, with incontinence of faeces and retention of urine. The next morning he had a very definite Kernig's sign and retraction of the head. At the end of 12 hours a profuse growth of meningococci was found in the fluid which earlier had appeared perfectly normal. At first it had appeared to be a case of measles. One may frequently obtain two or three perfectly clear fluids and finally one in which there is an abundant growth.

In some cases of hydrocephalus, the clear appearance and sterile nature of the fluid may lead to confusion. The fluid will appear perfectly clear with a high lymphocyte count. One such case appeared with a high leucocytosis. On the eighth day trephining showed that the roof of the fourth ventriculus was intensely adherent. The whole ventricular system was entirely shut off. About 2-1/2 drachms of spinal fluid were obtained. The meningococcus was grown from the ventricular fluid. This may serve as a kind of measure in estimating such cases. Since we know that in this case the entire ventricular system was shut off, this amount probably represents the contents of the theca alone. If 3-1/2 drachms are obtained it is probable that the entire ventricular system has been drained. When 3 drachms or under are obtained, the chances are that the case is one of hydrocephalus.

Typhoid and trench fever may also simulate cerebro-spinal fever. Retraction, Kernig's sign, and other symptoms may be found in such cases. Puncture shows the fluid to be sterile. There is no harm in puncturing a patient with typhoid or trench fever.

Cases may be classed according to types. The fulminating type needs no description. Death ensues within 24 hours. There is an acute type, with high temperature and very severe symptoms, which is usually fatal within four days. In such cases there is apparently no reaction of the body. The infection goes from bad to worse very rapidly. The brain and spinal cord are found to be intensely inflamed. There is another acute type which at first appears about the same, but which about the third day shows signs of improvement and reaction. The patient may get well with great rapidity.

There is a chronic type which clinically is unlike all the others. At the start there are hardly any symptoms of the disease, but, upon puncture, the fluid is found to be thick and infected. The case grows gradually worse and is usually fatal between the 17th and 20th days. It does not develop hydrocephalus. When a puncture is made, the cord is found to be thickly coated and covered with pus. There is also a certain amount of pus about the base of the brain but no marked inflammation about the ventricles. In a case of this type no good can be done. The patient dies in a curious dynamic way and not as in hydrocephalus.

There are sometimes relapses, although they are not usually serious. When they are, an increased bacterial infection is probably indicated. In one case of relapse 5 cc. of the man's serum sufficed to cut short the return of the disease.

In fatal cases which survive the fourth day hydrocephalus is usually the sole cause of death. In one case the patient died of an inter-current hemorrhage on the tenth day with marked dilation of the ventricles, but with none of the symptoms usually associated with hydrocephalus. Clinically a case in which hydrocephalus supervenes, appears to be progressing satisfactorily, but suddenly the patient begins to waste very rapidly, and the retraction becomes more and more pronounced. Some of these cases recover. They are accompanied with agonizing headaches, which, for some reason, pass away; the patient then gains flesh and becomes absolutely well.

The speaker said that his own experience confirmed the statement made by Prof. Netter, in his book on the subject, that almost all who recover from cerebro-spinal fever recover completely, and, except the few who are made stone deaf by it — for whom there is no help — they return to the service. In some cases a condition closely resembling disseminated sclerosis develops during convalescence. In five or six cases weakness about the limbs and strangury of the bladder occurred. In all cases in which there has been partial paresis of one arm or of the facial muscles recovery has been complete.

The speaker agreed with the conclusions of Colonel Gordon with regard to the use of sera, but emphasized the fact that at first the use of lumbar puncture alone without the injection of serum gave better results by way of treatment than were obtained by the serum then available.

Since the Lister serum has been used the results have been much better. As a supplement to any treatment complete rest for a long time is essential.

Sir William Leishman remarked that fortunately there had never been any epidemic prevalence of meningitis along the British front. He did not consider, however, that this fact could be attributed altogether to the efficiency of administrative measures, although he would have liked to claim it for them. There had always been a certain number of cases in hospital, reaching their maximum generally in the months of February and March and falling off very decidedly in August and September. Some of the cases in the summer, however, were as grave as those in the winter months. Each successive year the incidence of the cases in winter had fallen off, and in the present year, although we were at the end of February when the maximum was usually reached, the customary rise in the number of cases had not occurred. The speaker then described the measures taken at the front, which he stated were carried out under much more difficult conditions than those described by Colonel Gordon among the troops in England, and where it had been impossible to carry out swabbing on so large a scale. Attempts to find carriers had of necessity been confined to the groups of men known to have been in closest contact with the patients. In view of the general results of these investigations, he, and many others, felt doubtful as to whether the enormous labor entailed upon the bacteriologist had been justified. It was impossible to have a sufficiently large number of laboratories and bacteriologists in the field to carry out the examination of contacts on the scale indicated by Colonel Gordon. And he doubted in the epidemic he had observed in France whether it would have made a great deal of difference in the number of actual cases. At any rate the number of carriers found among the close contacts that were swabbed were surprisingly low, and it was exceptional for any of them to develop the disease It might perhaps be true that the standard of efficiency in such bacteriological tests was not in general as high as some, but certainly most of the bacteriologists concerned were of a very high standard of excellence. In most cases the technique was faithfully applied, but it had seldom been possible to discover more than two or three per cent. of carriers. Most of the cases had been sporadic, and it was usually impossible to trace any connection between the cases.

There remained a great deal to learn about the ways in which the disease spread. The speaker agreed with Colonel Gordon that overcrowding was an important influence. In practice, however, it was very difficult to trace individual cases to any special overcrowding. There was naturally a great deal of overcrowding in billets in rest villages, but he pointed out that the buildings occupied are usually very far from wind-proof.

He had formed a good impression of the value of the spray in dealing with contact: it was difficult, however, to get exact information on its value. A large number of the sprays were now used in France, and in some instances figures were available that would indicate that they had helped in controlling the incidence of infectious diseases. At one base an experiment covering a whole year had been carried out. During the first half of the year none of the men segregated as contacts had been subjected to spraying; in the second half, all were given spray treatment of one-percent. sulphate of zinc, which was applied systematically three days a week for twenty minutes at a time. During the first half of the year when the spray was not used, there were 302 contacts exposed to meningitis, of whom eight subsequently developed the disease, a percentage of 2.6. During the second half of the year, when the spray was used, there were 127 contacts, of whom only 1 (0.7 o/o) contracted meningitis. Roughly speaking there were three times fewer cases among the contacts who were exposed to the spray. The second half of the year, however, was that part in which the disease is usually less frequent. A similar diminution was shown among the contacts, with other infectious diseases, with the exception of diphtheria.

Almost every available serum for meningitis had been tried, and, on the whole, the results had been very disappointing, although in individual series, treated by one or another serum, the results had at times been good. In other instances, it was thought that lumbar puncture alone would have given as good results as were here obtained by the use of serum. The speaker said that he had hopes of the monovalent sera which were now available for the various types of meningococcus infection, but he feared that much valuable time was often lost in commencing serum therapy.

The mortality among cases at the front had been disappointingly high in spite of all methods of treatment — somewhere between 35 o/o and 38 o/o. The speaker said that he had been greatly impressed by what Colonel Gordon had said of this new antiendotoxic serum, and that he trusted it might prove a great advance in the serum therapy of the disease. In conclusion, he congratulated Colonel Gordon on his very clear statement and on his work, which had been of such high value to all concerned in the control of this grave disease.

Major-General Sir John Rose Bradford, A.M.S., emphasized the importance of being on the lookout for the disease, so that lumbar puncture might be performed before it was too late. He said that personally he was inclined, by his clinical experience, to divide cases arbitrarily into four groups :

1. Those in which the signs of meningitis — cerebral or spinal — are clearly marked.

2. Cases resembling typhoid or pneumonia. In these diseases it is known that a difficulty in diagnosis may exist, as meningitis may occur as a complication.

3. A group in which any diagnosis is extremely difficult and baffling owing to the anomalous symptoms present. Not many errors arise from these cases, however, as meningitis naturally suggests itself at once as a possible diagnosis when the clinical picture is anomalous.

4. Cases in which a perfectly definite, recognizable clinical picture of some other disease seems to present itself. It may be German measles, rheumatism, myalgia, trench fever, or nephritis. The majority of cases in which serious mistakes are made, the speaker believed, belonged to this group. He had seen cases of this kind kept in a hospital for days and even weeks before the proper diagnosis of meningitis was made. The possibility of confusion between meningitis and the disorders here mentioned is not likely to occur to anyone unless the danger of confusion has been called to his attention. Such cases of meningitis are usually of slight severity, but they are numerous enough to be of considerable military importance. The speaker said he had seen a number of such cases which at first clearly appeared to be German measles. Perhaps the most striking instances were among cases diagnosed as nephritis. The speaker said he did not refer to cases in which nephritis was a complication of cerebro-spinal fever, but to those in which the onset of the disease was that of nephritis not necessarily of a severe type, and where the symptoms of meningitis were entirely absent at first. The meningeal symptoms usually appeared after a few days of illness and were then easily confused with those of uremia. There were also cases in which the illness runs what is known clinically as a septicemic course with a high fever but no signs whatever of meningitis. In one such case a clear-cut picture of meningitis developed after five weeks of illness of a septicemic character. These anomalous cases may be of considerable importance in the spread of the malady and should, in the opinion of the speaker, be taken into consideration, in any discussion, in addition to the spread of infection by carriers and by declared and obvious cases of the malady in its usual forms.

The papers on Suture read at the morning and afternoon meetings on February 16 are given in full. Those by Doctors Lemaitre, Duval, and Vaucher have already appeared in an advance supplement to the present number. The rest, presented by English surgeons and bacteriologists, are here printed in full.

